# Psychobiography Training in Psychology in North America: Mapping the Field and Charting a Course

**DOI:** 10.5964/ejop.v11i3.938

**Published:** 2015-08-20

**Authors:** Joseph G. Ponterotto, Jason D. Reynolds, Samantha Morel, Linda Cheung

**Affiliations:** a Fordham University at Lincoln Center, New York City, NY, USA; Aalborg University, Aalborg, Denmark

## Abstract

Psychobiography holds an important position in the history of psychology, yet little is known about the status of psychobiographical training and dissertation research in psychology departments. This brief report identified psychobiography courses throughout North America and content analyzed a sample of 65 psychobiography dissertations to discern the theories and methods that have most commonly anchored this research. Results identified few psychology courses specifically in psychobiography, with a larger number of courses incorporating psychobiographical and/or narrative elements. With regard to psychobiography dissertations, the majority focused on artists, pioneering psychologists, and political leaders. Theories undergirding psychobiographical studies were most frequently psychoanalytic and psychodynamic. Methodologically, a majority of the dissertations were anchored in constructivist (discovery-oriented) qualitative procedures, with a minority incorporating mixed methods designs. The authors highlight the value of psychobiographical training to psychology students and present avenues and models for incorporating psychobiography into psychology curriculums.

A long-standing definition of psychobiography is “the explicit use of formal or systematic psychology in biography” (Runyan, 1982, p. 201). More recently, this definition has been expanded upon as follows: Psychobiography is “the intensive life-span study of an individual of historic significance in socio-cultural context using psychological and historiographic research methods and interpreted from established theories of psychology” (Ponterotto, in press, p. 3). Psychobiography is a topical specialty and sub-discipline within psychology, and psychobiographical research methods employ primarily qualitative and historiographic research approaches (Elms, 1994, 2005, 2007; Schultz, 2005b), but also quantitative (Simonton, 1998; Wiggins, 2003), and mixed methods designs (Ponterotto & Reynolds, 2013).

By its very nature, psychobiography is interdisciplinary, drawing on the intellectual disciplines of history and psychology. From history, psychobiographers draw on historiographic, biographic, and hagiographic methods, and from psychology they draw on its theories of personality and human development across the lifespan (Elms, 1994; Ponterotto, 2014a; Runyan, 1982). Figure 1 locates psychobiography at the intersection of history and psychology along with the specialty of psychohistory. Whereas psychobiography focuses on the intensive study of an individual life in socio-cultural-historic context, psychohistory is concerned with broad psychological interpretations of significant events or groups in history (Hofling, 1976; Runyan, 1982). For example, whereas an intensive psychological study of Sigmund Freud would constitute a psychobiography, a study of the birth of psychoanalysis and the evolution of the Vienna Psychoanalytic Institute, would constitute a psychohistory.

**Figure 1:**
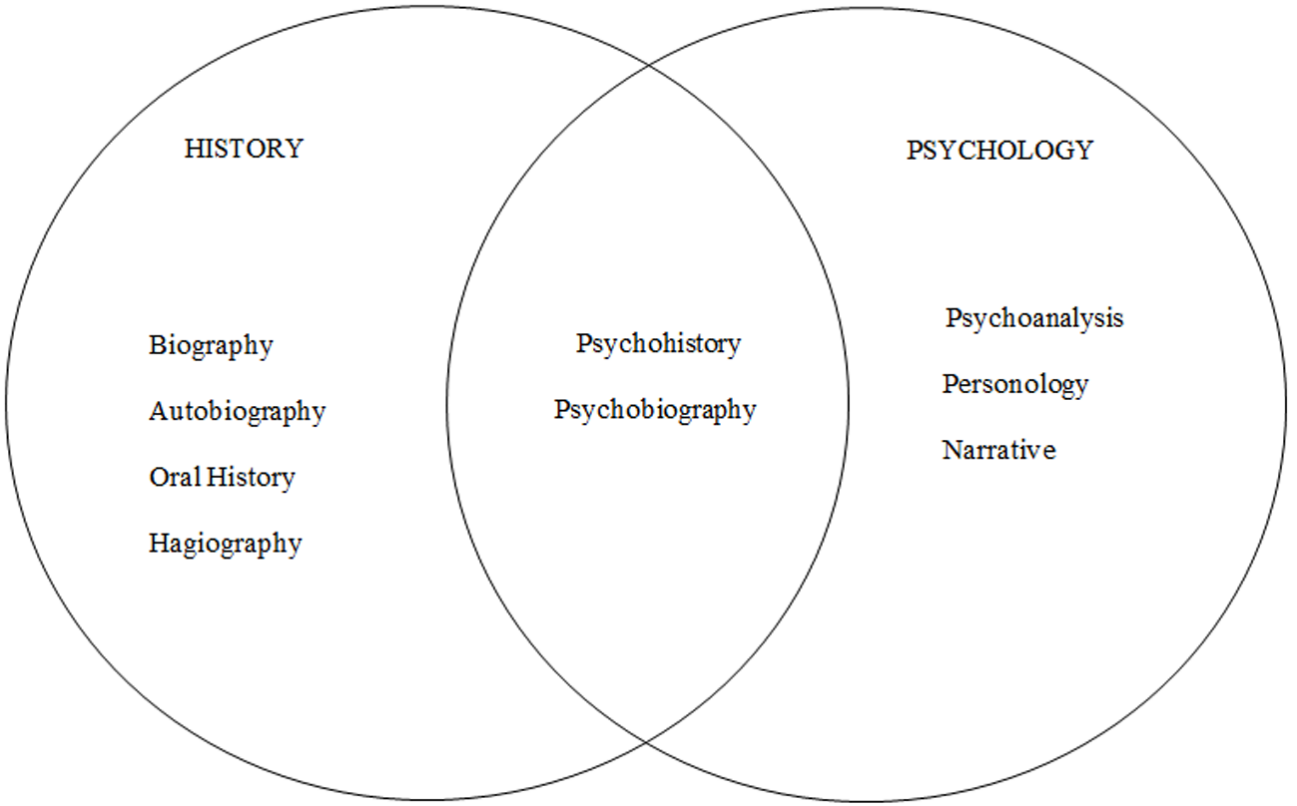
Locating psychobiography at the intersection of history and psychology.

The roots of psychobiography as a discipline in psychology have been traced to early work in biography and hagiography (the edifying study of saints and religious figures) (Kőváry, 2011; Mitchell & Howcroft, in press; Runyan, 1982). However, psychobiography became firmly established as a specialty in psychology when Freud (1910/1957) published his psychoanalytic profile of Leonardo da Vinci. Freud’s work at the turn of the 20th century ignited a strong interest among psychoanalysts in profiling historic figures throughout history (see Dooley’s 1916 review). In fact, psychoanalytic profiles of historic figures became so popular in the first three decades of the 20th century that the practice took on its own moniker, “applied psychoanalysis” (Runyan, 1988b).

By mid-20th century, the popularity of and production of psychobiography decreased as psychoanalysis fell out of favor in academic psychology, and as the positivist, experimental and quantitative emphasis took hold in psychology, particularly in Europe and North America (Runyan, 1982, 1988a, 1988b). During the second half of the 20th century, however, a renewed interest in psychobiography emerged, stimulated in part by the international impact of Erikson’s (1958, 1969) psychological profiles of Martin Luther and Mahatma Gandhi, respectively. Simultaneous to Erikson’s psychological profiling, other Harvard university psychologists such as Henry Murray (1967) and Gordon Allport (1965) were also studying the whole person and promoting the new specialty within personality psychology called personology, or the intensive psychological study of the individual person. Whereas psychobiographies most often focus on deceased “historic” figures, personology often emphasizes the intensive study of living persons who may represent more “average” individuals (Ponterotto, 2014a). The senior Harvard University professors along with their younger colleagues and students created a domino effect in promoting a resurgence of interest in narrative psychology generally, and psychobiography, specifically (Ponterotto, in press; Wiggins, 2003).

Systematic annual content analyses of psychobiography publications documented the trend of increasing interest and publication in psychobiography in both article and book form (Runyan, 1982, 1988a, 1988b, 2005; Schultz, 2005b). In recent years the first *Handbook of Psychobiography* (Schultz, 2005a) was published and there have been a slate of modern-day multi-theoretical psychobiographies of presidents, artists, writers, and a variety of intellectual giants written by psychologists in multiple specialty areas (e.g., Kasser, 2013; McAdams, 2011; Ponterotto, 2012; Schultz, 2011a, 2011b, 2013; Sharma, 2011). Furthermore, comprehensive reviews of psychobiographical research in the U.S., Europe, and South Africa have confirmed the increasing popularity of psychobiography internationally (Barenbaum & Winter, 2013; Fouché & van Niekerk, 2010; Howe, 1997; Kőváry, 2011; McAdams, 2001; Ponterotto, 2014a, 2014b). Presently, the *Journal of Psychology in Africa* is devoting a whole special issue (October, 2015) to psychobiography (Fouché, in press).

Recently, in tracking the “narrative turn” (Laszlo, 2008) in personality psychology research over the past two decades, Kőváry (2011, p. 739) declared that the psychology field is currently in the midst of “a renaissance in psychobiography.” At this time, however, it is not clear whether this resurgence in psychobiography interest is reflected in academic training programs across psychology departments in North America. Questions that need to be addressed include: Do psychology departments offer a course in psychobiography? Might psychobiographical theory and methods be integrated into traditional core courses in psychology, such as “Personality Psychology” or the “History of Psychology”? How do students and faculty feel about the importance and relevance of psychobiography to their training in psychology? Are doctoral students in psychology conducting psychobiographical dissertations, and if so, what psychological theories and research methods are anchoring these dissertations?

One modest goal of the present study is to begin to map the current status of psychobiography training in psychology through an assessment of course offerings in North America and through the identification of specific Psychology Departments offering such courses. A second goal is to examine a sample of psychobiography dissertations to ascertain which departments/universities are supporting such research and to identify the theories and methods used to guide the dissertations as well as the nature of historical subjects studied. A final goal is to provide some direction and ideas for faculty and students interested in expanding their department’s training in psychobiography.

## Method and Procedures

The method for this study involved three distinct components: random sampling of psychology departments in North America; purposeful listserv and snowball sampling of professors who teach in the psychobiography area; and a review of psychobiography doctoral dissertations. This study was approved by the authors’ home Institutional Review Board.

The method and procedure for each component of the study is described next.

### Random Sample of North American Universities

To gather a general sense of the extent to which formal psychobiography courses are available in psychology curriculums, we examined a random sampling of 100 universities in the United States, and 10 randomly selected Canadian universities, to see if they offered a course in psychobiography. Given there are roughly 2000 colleges and universities in the United States (The University of Texas at Austin, 2015a) and 100 in Canada (“List of Universities,” 2015), our sampling represented 5% of U.S. institutions and 10% of Canadian institutions.

U.S. institutions were selected based on a stratified random sample from the “List of U.S. Universities by State” (The University of Texas at Austin, 2015b). One large state and one large private university from each of the country’s 50 states were identified for review. Canadian institutions were randomly selected from the “International Colleges & Universities” website (http://www.4icu.org/ca). Selection criteria required that the psychology course listings and descriptions were available online. Specialty universities such as art or music schools, medical schools, photography schools, among others were not included.

Once appropriate institutions were identified, the authors examined the list and descriptions of all courses offered in the undergraduate and graduate psychology curriculums. First, the curriculums were examined to locate any courses specifically in psychobiography [or psychohistory, lifestory, or narrative psychology]. Second, the traditional core courses of “Personality Psychology,” and “History of Psychology,” which are more likely to incorporate psychological biography or lifestory methods, were also reviewed to see if any included psychobiographical components in their course descriptions. Finally, courses on Research Methods or Qualitative Research were also examined to determine if the courses included psychobiographical research methods as a component of the course. Clearly quantitative courses with titles such as Experimental Psychology or Quantitative Research Methods were excluded. If links to course syllabi were available on university websites, we examined the syllabi for psychobiographical content. The frequency of relevant offerings and the specific professors and departments were logged.

### Purposeful Listserv and Snowball Sampling

Anticipating that there would be few psychobiography courses offered in a random sampling of psychology curriculums, the authors incorporated purposeful listserv sampling in an effort to identify courses in psychobiography and the professors who taught them. More specifically, we sent a brief course survey (described below) to listservs or discussion groups affiliated with the following Divisions of the American Psychological Association (APA): 2 (Teaching of Psychology), 8 (Personality and Social Psychology), 17 (Counseling Psychology), 44 (Lesbian, Gay, Bisexual, and Transgender Issues), 52 (International Psychology), and 5 (Division of Quantitative and Qualitative Methods in Psychology’s qualitative interest group). We also sampled the listserv associated with the Association for Research in Personality. Each of these groups were open to using the listserv to send out a brief survey on “psychobiography in the curriculum.” Other listservs we investigated were not open to research participation queries.

In addition to the listserv sampling, we also identified potential professors of psychobiography and related courses through the qualitative procedure known as “snowball sampling” (Patton, 1990). Individuals known to work and publish in the psychobiography area through a literature review were contacted by email and also asked to recommend other colleagues they knew working in psychobiography.

For professors responding to the listserv inquiry or the snowballing efforts, they were sent a brief five-question open-ended survey to gather specific information on their relevant course and to ask their opinions on the relevance and importance of such courses. The survey questions were as follows:

1. Please specify the name of the course; whether it is an elective or required course; undergraduate, graduate, or open level course; and taught on-campus, on-line, or hybrid.
2. How has the course been received by students and your department faculty?
3. How is this content area and its relevant methodologies/approaches useful to the training of psychologists and other social and behavioral scientists?
4. From your perspective, where do psychobiographical and/or narrative methods fit within the field of psychology?
5. If your department offers master’s or doctoral degrees, would a “Psychobiography Proposal” be approved for a master’s thesis and/or a doctoral dissertation?

### Dissertation Review

In an effort to examine original research in psychobiography generated by graduate students, we content-analyzed a sample of psychobiography doctoral dissertations. To locate doctoral dissertations focusing on psychobiography, we entered the word “psychobiography” in the “title,” “abstract,” or “keyword” search categories of the ProQuest dissertation on-line catalogue. Initially, 72 dissertations were identified of which 65 met our criteria of being written in English and being completed at institutions in the United States and Canada. We developed a content analysis template and coded each dissertation in the following 13 categories: Author, year, title, biographical subject, profession of subject, major contribution of subject, life space of subject, length of dissertation, mentor/chair of dissertation, university, university department, anchoring psychological theories, and research methods employed.

To ensure consistency of coding, the first three co-authors of this study proceeded as follows. First the team worked together on five randomly selected dissertations from the larger pool of 65 to decide on and practice the coding procedures. Subsequently, the senior author (who is more versed in psychobiography) completed coding a set of eight dissertations, four of which were also coded independently by the second author, and the remaining four which were also coded by the study’s third author. The team then met to examine consistency across both sets of coded dissertations. Consistency was generally quite high, with minor differences in the amount of detail presented when coding theories undergirding each study and in coding the variety of research methods employed. After discussion and consensus gathering, the remaining dissertations were divided among the three authors. The team would then meet once a week to discuss and clarify any confusion in coding. A supplemental reference list of the 65 Psychobiography Dissertations reviewed in this study is available from the senior author.

## Results: Mapping the Field

### Psychobiography Courses in the Curriculum

The first goal of our analysis was to map psychobiography training nationwide in terms of the extent and locales of psychobiographical training. The random survey of 110 psychology departments in North America revealed zero courses specifically in psychobiography, and 12 courses that though not named psychobiography, incorporated psychobiographical elements into the design of the course. Through our second procedure, snowball and list-serve sampling, 28 professors responded indicating they taught in the area of psychobiography or knew of colleagues who did so. Of this group, 17 Professors completed the five-question course survey.

Table A1 lists the psychobiography and related courses identified through both sampling methods. Only six courses with “psychobiography” in its title were located, and all were targeted for upper-division undergraduate audiences. An examination of six psychobiography syllabi submitted led us to develop an integrative version that is available from the senior author.

An additional 48 courses were located (12 through the random survey and 36 through the listserve and snowball sampling) that appeared directly related to psychobiography and/or required psychobiography readings and assignments. These courses are listed in Table A1 along the categories of personality psychology, narrative and life story, culture, biography, and autobiography, history of psychology, genius and creativity, political psychology, and “other” content areas (see Table A1).

### Open-Ended Survey

Responses to the open-ended survey questions were examined using basic phenomenological theme analysis (Creswell, 2013). Meaning unit (sentences or full paragraphs) responses for each of the five questions were extracted and reviewed for common themes within each of the five questions. Representative quotes were chosen to highlight participant voice on each question or each subtheme within the broader question.

Examining the results of the survey along the five questions posed reveal the following highlights: 1) The majority of the courses offered were elective, taught on campus (rather than on-line) and targeted for upper-division psychology majors or graduate students. 2) All 17 professors responding indicated that the course was very well received by students, and generally admired by faculty colleagues. Sample quotes in this vein include:

“Students have absolutely loved it – some of the best teaching evaluations I have ever received. The faculty have also been happy with it.”

“The course has been very well-received. The faculty in the Critical Social/Personality psychology program value lives, narrative, and psychobiography as critical to our work as social scientists.”

“The psychobiography component has been well received. The most common comment I get is ‘Why don’t more psych courses talk about this,’ and ‘Why isn’t there more research employing this approach’?”

“Psychobiography has been really well received by students, my department, and the college! I taught it as an elective in the department before being encouraged by students and the director of the honor’s college to make it an honor’s course. The students enjoy it and my colleagues appreciate the engagement students get with theory at a practical, immersive level.”

“I received positive feedback from students on the psychobiography component in lab; several of my colleagues reported to me that their students (advisees or common students) reported positively about that component as well.”

A minority of respondents (two of the 17) noted that their students responded very well to the course, but that many fellow faculty members, though supportive, were unaware of psychobiography and narrative approaches. For example,

“In my own research lab, students received it well. Most faculty members, however, do not know or understand narrative theory or method in general and psychobiography in particular.”

“Student evaluations of the course are consistently positive. Department faculty do not really know enough about the course to evaluate it.”

3) All respondents thought the content area and methodologies of psychobiography are critical to professional development of psychologists and other behavioral scientists. Responses to this question clustered in three areas. First, respondents believed that psychobiography and other narrative approaches in psychology increased students’ understanding of themselves and helped them understand psychological theories as applied to individual lives. Second, such methods promoted the understanding of qualitative research approaches and the complementarity of qualitative and quantitative approaches in an in-depth understanding of an individual in social context. Third, psychobiographical and narrative methods were particularly salient to students wishing to practice as mental health professionals and held relevance for others specialties in psychology. Below, we include quotes from these areas separated into six content areas.

#### Self-Understanding and Theoretical Application

“I first used lifestory method to teach my students in my research lab how to conduct in depth interviews to explore cultural identity development. Students struggled with analyzing the data collected. I found psychobiography was a helpful tool for the students to examine their own lives and gain deeper insights.”

“It makes students grapple with a number of important questions – e.g., how well do psychological theories and concepts work to understand individuals? Can we use them without pathologizing or labeling the individual, or reducing his/her behavior to some simplistic explanation? What are the many different factors we need to consider if we want to understand a person?”

“I think this focus is useful on two fronts. First it forces students to engage with theories and concepts in a more concrete manner. As Allport said, idiographic research really represents the proving ground for psychological theories. Second, it is useful because I believe that one of the major goals of personality psychology is to describe individual lives.”

#### Promotes Qualitative Research Skills and Complementary Research Approaches

“Students leave this course with a solid grasp of interviewing and other qualitative methodologies. They also practice using a number of interpretive strategies for interpreting individual life stories in the pursuit of psychologically relevant research questions to better understand how individuals internalize and/or resist larger social structures, policies, and life challenges. The focus on ethics and ‘narrative relationships’ is also central to all doctoral students in psychology.”

“Psychobiography is, in my mind, a critical tool for training at any level. At an advanced level, I see psychobiography as part of a cycle of the research process and an important part of determining how theory and quantitative data play out in an individual lived life. By deeply immersing ourselves into how theory and data are manifest in one person, we can generate new, creative questions broadening and deepening both theory and Research. It is also incredibly useful for undergraduate learning as it gets students working with theory and exploring data in a creative, fun way that they generally enjoy engaging with more than just a traditional research paper. They put theory and research to practical use to understand someone they are interested in which stimulates their research skills, catalyzes creative thought, and facilitates at a deeper level than what would happen in traditional undergraduate pedagogy.”

“I like the idea of different layers of research and understanding within personality and examining the stories people tell are definitely a useful way of understanding them. I also think it is helpful for psychologists (and others) to take a step back from the tightly controlled experiments and examine the real lives of people. If your aim is to understand people, then ignoring the utility of the narrative approach is ignoring a huge part of who a person is.”

“The course is highly relevant, as it widens students’ perspectives to the variety of epistemological positions and research methods available within the broad field of psychology. It reminds students that psychology is grounded in the study of individual people. It gives them an approach for understanding individuals that augments the methods from other psychological approaches.”

#### Relevance to Clinical Training

“One of the things clinicians do is listen to life stories and attempt to help clients make sense of the stories. Life stories illustrate personality development, helping future researchers and practitioners as well to learn how to take a life narrative and how to use it with their clients or research participants is useful for training.”

“The approach taken in this course helps students develop an appreciation for the history of the discipline, and how that history informs and shapes current theory and research in one area of personality study. In addition, I feel that understanding how people narrate their lives is an important tool for clinical psychologists and counselors to have (most of our students aspire to be mental health professionals). Furthermore, there is a strong emphasis in the course on psychobiography as a scientific enterprise, one that uses rigorous methods and psychometrically sound measures to study life narratives.”

4) Most respondents believed that psychobiography and related topics (narrative knowing) are central and critical to the field of psychology. Responses to this question clustered into three areas: the importance of studying the individual life in socio-cultural context, the importance of psychobiography and narrative as approaches to balance traditional quantitative study, and specific specialties in psychology where these methods seem particularly important.

#### Individual Life in Cultural Context

“The study of individual lives should be at the center of psychology, so psychobiographical and/or narrative methods should be too; these methods can be used in other kinds of studies as well, once we realize how important it is to consider the many factors that shape people’s experience and their views of themselves and the world.”

“I tend to think of narrative less in terms of discrete methods and more in terms of conceptualizing human lives and persons …. Narrative knowing is at the very heart of the field of psychology. Much of psychology simply doesn’t know it yet!”

“[These approaches] sit at the nexus of epistemological approaches, blending both paradigmatic and narrative (to use Bruner’s terms) worlds. They provide an option for studying personality, development, and culture.”

“Psychobiographical method is a helpful tool for students to gain a deeper understanding of how culture operates in their own lives; this in turn impacts how they can carry out research and clinical practice. Narrative analysis has been a fruitful way to conduct research on cultural identity development.”

#### Supporting Quantitative Approaches to Studying Lives

“I see psychobiography and other related narrative methods as an essential part of the testing and building of theory that should be paired with quantitative data. By bringing what we know from quantitative data and theory to the life or lives of an individual we can not only gain more insight into the individual, which is in and of itself a useful contribution, but can also discover new directions to take in theories and in quantitative data collection. Therefore, I see such methods as highly generative and essential pieces to the empirical process in psychology.”

“I believe that it is central and can be very much in dialogue with other approaches to understanding human behavior. Most centrally, psychobiography enables us to examine the degree to which more nomothetic hypotheses and ‘findings’ particularize to the life story of an individual person. It allows us to more fully engage the emotion of ‘persons-in-context’ and lives and structures.”

#### Relevance to Other Specialties in Psychology

“I think that narrative methods operate primarily within developmental and personality psychology and to a lesser extent within social and cognitive psychology, much of clinical is more or less narrative and psychobiographical but does not necessarily identify as such.”

“I see these methods closely tied to clinical, social, personality, developmental, industrial/organizational, and evolutionary psychology.”

“I would say personality, though there is a clear connection to clinical/counseling, and health research.”

5) Finally, with regard to whether departments allowed psychobiography master’s theses or doctoral dissertations, 10 of the 17 respondents were in graduate degree-granting institutions. Of this subsample, six responded that psychobiography projects were acceptable, one said no, and three responded that psychobiography theses and dissertation would be acceptable if combined with other research approaches, for example:

“A dissertation focused on psychobiographical research but included additional complementary methodologies (qualitative and/or quantitative) would absolutely be acceptable and recognized as an important contribution.”

“Psychobiography would be acceptable for a doctoral dissertation, but we have a commitment to multimethod education, so students are likely to use this approach in conjunction with others in their research.”

### Psychobiography Doctoral Dissertations

With regard to the 65 dissertations reviewed, the overwhelming majority (80%) were conducted to complete final requirements for the Ph.D. degree, while a minority (18%) fulfilled Psy.D. requirements and the Doctor of Nursing Science (2%). The dissertation copyright dates ranged from 1978 to 2014. In terms of production trends over time, results indicated 2% appeared during the 1970s, 20% during the 1980s, 40% in the 1990s, and 38% in the 2000s. Sixty percent of the dissertations were completed in the more traditional North American “university,” whereas 40% were conducted in “professional schools” (e.g., California School of Professional Psychology, Wright Institute). Further, 74% of the studies were conducted in Departments of Psychology, with the remainder (26%) spread out across various departments and disciplines or combinations of disciplines (e.g., history, counseling, religion, human and organization systems, joint program in psychology and religious studies).

The dissertations were advised by a wide array of mentors, with four scholars mentoring multiple psychobiography dissertations: George Stricker at Argosy University, Peter M. Newton at the Wright Institute, Hilary E. Bender at the Massachusetts School of Professional Psychology, and Stephen J. Hobbs, formerly at the Wright Institute and now in Private Practice. The most prolific mentor of psychobiography dissertations in out sample was Dr. Newton with five.

Data analysis revealed geographic trends in the production of psychobiography dissertations. The majority emanated from the west coast (44%) and the northeast (34%), with smaller output in the midwest (14%), south (3%), mountain region (2%), and from Canada (3%). U.S. states particularly well represented in psychobiographical dissertation research were California (44%) and New York (20%), with New Jersey and Ohio following along at 6% each. Academic institutions producing the most psychobiographical dissertations were the Wright Institute (n = 8), the California Schools of Professional Psychology (n = 6), and Adelphi University’s Derner Institute for Advanced Psychological Studies (n = 6).

Psychobiography, like its cousin biography, often focuses on deceased historic and public figures (see Elms, 1994; Runyan, 1982). The present data supports this trend as all but two dissertations focused on deceased individuals. One dissertation focused on a living and incarcerated serial killer (Joel D. Rifkin; by DeSantis, 2002), and one on a fictional character (Horace Benbow; by Meixner, 1992). Of the historical figures, 32% were born in the 20th century, 56% were born in the 19th century, while the remainder (12%) were born during the 18th century or before. The subjects ranged in life span from 28 years (psychobiography of Egon Schiele; by Walrod, 1978) to 94 years (psychobiography of Frances Crosby; by Albertson, 1992), with a mean age at death of 64 years. With regard to gender of historical subjects, the majority were male (63%) versus female (32%), with the remaining focused on multiple figures or an entire family (5%).

The careers or notable contributions of the psychobiographical subjects could be partitioned into the following six areas: various artists (51%, e.g., sculptors, painters, novelist, poets, and playwrights), psychologists (14%), political leaders (9%), religious healers/leaders (6%), incarcerated adults (3%), a diverse miscellaneous group (8%), and an “undefined” career group (9%).

In terms of theoretical lenses anchoring the psychobiographical research, the majority were either traditionally psychoanalytic (32%), object relations focused (11%), or other dynamic variants (8%); while Levinson’s (12%) adult developmental model and Erikson’s (11%) psychosocial model were also popular. Less frequent among theoretical anchors were humanistic/existential models (3%; e.g., Bugental). Finally, roughly 23% of the dissertations used a scattering of other theories (e.g., family systems, racial identity, feminism, Big Five). Interestingly, 60% of the psychobiography dissertations were anchored primarily in a single theory, while 5% relied on multiple theories within the same school of thought, and 35% relied on multiple theories representing different schools of psychology.

A wide variety of research methodologies were employed in the conduct of the psychobiographical research. Historiographic and qualitative methods predominated and included archival document reviews (including biographies/autobiographies, letters, diaries, photos, recorded interviews, speeches, and creative productions [analysis of drawings, poems, films, paintings]). In the case of the living subject (serial killer, Joel D. Rifkin) and more recently deceased subjects, recorded interviews with subject and/or surviving friends and relatives were incorporated. It would be fair to say that qualitative research anchored in the emergent, discovery-oriented constructivist-interpretivist paradigm (see Guba & Lincoln, 1994) was most prominent among the studies.

Finally, a minority of the dissertations also included a post-positivist element by incorporating some quantitative analysis and thus served as mixed-methods designs. For example the study of Joel D. Rifkin included document analysis, personal interviews, and the analysis of a wide spectrum of objective and projective personality tests as well as tests of intelligence (DeSantis, 2002).

## Discussion: Charting a Course

The present limited study identified few psychobiography courses offered in psychology departments nationwide. A larger number of courses were anchored in psychobiography and/or incorporated psychobiographical elements (e.g., readings, assignments), but were not specifically marketed as courses in “psychobiography.” It is somewhat surprising that given the historic and influential role of psychobiography to the fields of personality, human development, psychological theory, and individual differences (including exemplar model of “genius,” “goodness,” and “evil”) that more attention to psychobiography as both a topic and method is not extant. Complimenting study in psychobiography, there were a number of professors who incorporated more general personologic and narrative research approaches in the curriculum. Particularly influential in this regard was McAdams’s (1993) *The Stories we Live by*.

Respondents to our five-item open-ended survey where in unanimous agreement that psychobiography and other narrative approaches to studying individual lives was central and critical to training in psychology. Such approaches equip students in psychology with critical skills in self-understanding and cultural awareness, in applying theories of psychology to whole lives in historical context, in understanding the importance of working with qualitative data in addition to the traditional quantitative emphasis, and these approaches have direct relevance to enhancing clinical skills for practitioner-oriented students. With regard to psychobiography, the texts of Elms (1994), Runyan (1982), and Schultz (2005a) were deemed as essential training for all psychologists. Similarly the life story method outlined in McAdams (1993) was identified as required reading for all psychology students.

The present study was able to identify a good number of doctoral dissertations focused on psychobiography. In fact, the output of dissertations seemed disproportional to the few psychobiography courses identified, and therefore it may be likely that doctoral programs promoting psychobiography as a dissertation topic may not offer actual “psychobiography” courses in the curriculum. This raises some concern in that doctoral students may be pursuing a complicated methodology in their dissertations without formal training in the procedure. It is likely that students are learning psychobiography on their own or may have mentors qualified to supervise their work. Alternately, our limited sampling procedures may have missed professors working in departments where psychobiography training is well integrated.

Certainly the benefits of training students in psychobiographical research merits increased discussion on the place of psychobiography and other narrative approaches in the curriculum. Among the benefits of training in psychobiography are understanding the individual person in historic and cultural context, mastering multiple psychological theories, emphasizing the complementarity of quantitative and qualitative research methods, and gathering knowledge in related disciplines of history, journalism, and political science (Elms, 1994; Howe, 1997; Kőváry, 2011; Ponterotto, 2013, 2014a, 2014b; Runyan, 1982; Schultz, 2005a).

Perhaps the most comprehensive approach to psychobiography mastery is the development of full courses on the subject, along with promoting and supervising psychobiography research studies at both the undergraduate and graduate levels. As noted in Figure 1 discussed earlier, psychobiography is an interdisciplinary endeavor and psychology programs can consider joining with other departments such as history, political science, and journalism to offer joint courses and thus drawing on the expertise of interdisciplinary faculty. An alternate, though less comprehensive route, may be to embed psychobiography into pre-existing core psychology courses and including relevant readings and projects. Natural nominees for such courses would be personality, human development, history, research methods, political psychology, the psychology of the gifted and talented, and forensic psychology. A number of courses in Table A1 follow this model.

The psychology profession, with its momentum toward methodological diversity (e.g., Gergen, Josselson, & Freeman, 2015; Josselson, 2013), can advance with more doctoral level research in psychobiography. The present study found roughly one-half of psychobiography dissertations being generated by traditional universities, and the remainder by professional schools and institutes. Perhaps psychology departments in traditional universities can partner with colleagues in professional schools to share resources and expertise and provide doctoral (and masters) students with expanded research options. Kőváry (2011) is convinced that the psychology profession, internationally, is in the midst of a renaissance of psychobiography; it will be interesting to see how this prediction unfolds in North America in the coming decade.

Naturally, the present brief report has marked limitations in terms of design and generalizability. The random sampling of universities was limited though reaching all 50 states in the United States as well as major universities in Canada. From the dissertation component of the study it was clear that a good amount of psychobiographical research is being conducted in non-traditional universities, such as Institutes and Professional Schools of Psychology, which were not included in the random survey component of this study. Therefore it is likely that there may be more psychobiography courses offered in some of these institutions. The convenience sampling procedure relied on snowballing procedures and select list-serve e-mail requests, and the random sampling was limited to large universities in North America. It would be important in follow-up research to randomly select a large sample of university and professional psychology curricula in Europe and world-wide to evaluate with certainty whether and where psychobiography courses are offered. It would be of value to assess (using quantitative surveys, focus groups, and in-depth personal interviews) both student and faculty opinions on the place and quality of psychobiographical training in their respective departments. Our hope is that this study stimulates discussion in psychobiography among undergraduate and graduate departments of psychology. Kőváry (2011, p. 739) claims that the psychology profession is in the midst of a “renaissance in psychobiography.” It will be important to test this prediction worldwide and examine how a resurgence in psychobiography is manifested in psychology training programs and in publications.

## Appendix

**Table A1:** Psychobiography Courses and Courses Incorporating Psychobiographical Training*

Specific Courses with “Psychobiography” in Title
Pacific University Oregon, Forest Grove Campus, PSY-444, “Psychobiography,” William Todd Schultz, Department of Psychology, Upper Division Undergraduate Level Amherst College, Amherst, MA, PSYCH-38, “Psychobiography: The Study of Lives,” Amy P. Demorest, Department of Psychology, Upper Division Undergraduate Level University of the South, Sewanee, PSY-406, “Psychobiography Seminar,” Nicole B. Barenbaum, Department of Psychology, Upper Division Undergraduate Level Monmouth University, “Psychobiographical Research,” Frances K. Trotman, Hybrid Elective course Canisius College, Undergraduate Honors 270, “Psychobiography,” Jennifer Lodi-Smith Marquette University, “Psychobiography,” Ed de St. Aubin, Department of Psychology, Undergraduate Level
Specific Courses Anchored in (or Incorporating) Psychobiography
Personality-Oriented Psychology Russell Sage College, Troy, NY, “Personality Theory” (Persons in the Personality Theory), Susan C. Mueller, Department of Psychology, Upper Division Undergraduate Level (see Mueller, 1985) Amherst College, Amherst, MA, PSYCH-338, “Personality and Political Leadership,” Amy P. Demorest, Department of Psychology, Upper Division Undergraduate Level Rutgers University, New Jersey, “Seminar in Personality Psychology,” Dan Ogilvie, Department of Psychology, Graduate Seminar Level Rutgers University, New Jersey, Psych 16-830:541, “Seminar in Personality Psychology,” Dan Ogilvie, Department of Psychology, Upper Division Undergraduate Level Simon Fraser University (Canada), PSYC 370, “Theories of Personality,” Bob Ley, Department of Psychology University of North Carolina at Chapel Hill, Psychology 501, “Advanced Personality,” Department of Psychology Duke University, PSY 629S, “Social Behavior and Personality,” Rick Hoyle, Department of Psychology Canisius College, PSY 302, “Personality Psychology,” Jennifer Lodi-Smith, Upper Division Undergraduate Course University of Prince Edward Island, “Theories of Personality,” Scott Greer, Department of Psychology DePauw University, “The Psychology of Personality” (with lab), Ted Bitner, Department of Psychology Marquette University, “Personality,” Ed de St. Aubin, Department of Psychology, Clinical Psychology Program University of Dayton, “Personality,” Jack Bauer, Department of Psychology University of British Columbia, “Personality Psychology,” Katherine H. Rogers, Department of Psychology, 300-Level Undergraduate Elective University of California at Riverside, PSYC 226, “Theories and Concepts in Personality Psychology,” Will Dunlop, Required Graduate Course University of California at Riverside, PSYC 150, “Personality Psychology,” Will Dunlop, Undergraduate Elective Course Northeastern University, “Personality Research Methods,” Randy Colvin, Department of Psychology John Jay College of Criminal Justice, PSYC 74003, “Historical and Theoretical Foundations of Critical Social/Personality Psychology,” Susan Opotow, Department of Psychology
Narrative and Life Story-Oriented Psychology University of California at Berkeley, “Life Histories and Case Studies,” William McKinley Runyan, School of Social Welfare LeMoyne College, Syracuse, New York, PSY 444. “Story in Psychology: Narrative Perspectives on Human Behavior,” Vincent W. Hevern, Department of Psychology, Upper Division Undergraduate Level College of the Holy Cross, “Life and Literature,” Mark P. Freeman, Psychology Department, Undergraduate Level College of the Holy Cross, “Time, Memory, and the Life Story,” Mark P. Freeman, Undergraduate Course in First-Year Program Kalamazoo College, PSYC 430, “Interviewing and Narrative Analysis,” Psychology Department Olin College, PSYCH 221/ AHSE 2199, “Narrative Psychology,” Jonathan Adler, Psychology Department, Undergraduate Elective Course Lewis and Clark College, CPSY 590, "Narrative Therapy," Steve Berman, Graduate School of Education and Counseling City University of New York (CUNY) Graduate Center, PSYC 80102 (27651), “The Study of Lives,” Jason VanOra, Interdisciplinary course for Critical Social/Personality, Environmental Psychology, and Social Welfare Programs, Doctoral Level Marquette University, “Narrative Psychology,” Ed de St. Aubin, Department of Psychology, Advanced Undergraduate Level Boston Graduate School of Psychoanalysis, “Narrative Research,” Stephen Soldz The University of Chicago, CHDV 28900 (PSYC 27600, SOSC 28900), “The Study of Lives: Psychology and Biography,” Bertram J. Cohler
Culture, Biography, and Autobiography Smith College, Psychology 333, “Identity through Psychology, Fiction, and Autobiography,” Bill E. Peterson, Elective Undergraduate Senior Seminar Course, Undergraduate Course in First-Year Program Arizona State University ENG 420, “Multicultural Autobiographies”, Thomas V. McGovern Arizona State University, IAS 220, “Psych/Multicultural Narr/Relig,” Thomas V. McGovern Rice University, RELI 430/584, “Religion, Psychology, & Culture,” William Parsons, School of Humanities, Department of Religious Studies Morgan State University, ENGL 473, “Literary Biography and Autobiography,” English Department, Undergraduate Elective Course Fuller Graduate School of Psychology, “Cultural/Spiritual Narratives in Psychology,” Jenny H. Pak, Doctoral Course in Clinical Psychology Fuller Graduate School of Psychology, “Narrative, Culture, and Attachment in Psychotherapy,” Jenny H. Pak, Clinical Psychology Program, Elective Graduate Psychology Course University of Minnesota, PSY 1905-001, “The Cultural Psychology of Storytelling,” Moin Syed, Department of Psychology, Freshman Seminar Course
History-Oriented Psychology 1. University of California at Davis, Psych 185, “The History of Psychology,” Dean Keith Simonton, Department of Psychology, Undergraduate Level University of California at Davis, Psych 220, “The History of Psychology,” Dean Keith Simonton, Department of Psychology, Graduate Level Fordham University Graduate School of Education, PSGE 6615, "History and Systems of Psychology," Joseph G. Ponterotto, Required Course for PhD in Counseling Psychology
Genius and Creativity University of California at Davis, Psych 175, “Genius, Creativity, and Leadership,” Dean Keith Simonton, Department of Psychology, Upper Division Undergraduate Level Concordia University – Portland, “Beautiful Minds: A Seminar Course on the Psychology of Genius,” Kevin E. Simpson, Department of Social Sciences, Open to All Psychology Majors
Political Psychology University of California at Los Angeles, M236A/M261A, “Proseminar: Political Psychology”, David O. Sears University of North Carolina at Chapel Hill, POLI 417, “Advanced Political Psychology,” Timothy J. Ryan, Department of Political Science, Undergraduate Elective
Other Course Content Areas Rutgers University, New Jersey, “Abnormal Psychology,” George Atwood, Department of Psychology, Upper Division Undergraduate Level College of the Holy Cross, “Time, Self, and the Good Life,” Mark P. Freeman Fordham University Graduate School of Education, PSGE 7680, “Qualitative Research Methods in Counseling Psychology,” Joseph G. Ponterotto, Required Course for PhD in Counseling Psychology University of Dayton, “Adult Development and Aging,” Jack Bauer, Department of Psychology University of Texas Austin, “Psychology of Literature Class,” Jennifer Beer, Department of Psychology, Undergraduate Level

**Note*. Some of these courses are not offered at the current time or are now taught by different professors.
